# Association between Venom Immunotherapy and Changes in Serum Protein—Peptide Patterns

**DOI:** 10.3390/vaccines9030249

**Published:** 2021-03-12

**Authors:** Joanna Matysiak, Eliza Matuszewska, Marek L. Kowalski, Sławomir W. Kosiński, Ewa Smorawska-Sabanty, Jan Matysiak

**Affiliations:** 1 Faculty of Health Sciences, The President Stanisław Wojciechowski State University of Applied Sciences in Kalisz, 62-800 Kalisz, Poland; jkamatysiak@gmail.com; 2 Department of Inorganic and Analytical Chemistry, Poznan University of Medical Sciences, 60-780 Poznań, Poland; eliza.matuszewska@ump.edu.pl; 3 Department of Immunology and Allergy, Medical University of Lodz, 92-213 Łódź, Poland; marek.kowalski@csk.umed.lodz.pl (M.L.K.); slawomir.kosinski@csk.umed.lodz.pl (S.W.K.); ewasm@csk.umed.lodz.pl (E.S.-S.)

**Keywords:** *Hymenoptera* venom allergy, MALDI-TOF MS, protein–peptide profiling, proteomics, venom immunotherapy

## Abstract

Venom immunotherapy (VIT) is administered to allergic patients to reduce the risk of dangerous systemic reactions following an insect sting. To better understand the mechanism of this treatment and its impact on the human organism, we analysed serum proteomic patterns obtained at five time-points from *Hymenoptera*-venom-allergic patients undergoing VIT. For statistical analyses, patients were additionally divided into two groups (high responders and low responders) according to serum sIgG4 levels. VIT was found to be associated with changes in seven proteins: the fibrinogen alpha chain, complement C4-A, complement C3, filamin-B, kininogen-1, myosin-9 and inter-alpha-trypsin inhibitor heavy chain H1. The number of discriminative *m/z* (mass-to-charge ratio) features increased up to the 90th day of VIT, which may be associated with the development of immunity after the administration of increased venom doses. It may also suggest that during VIT, there may occur processes involved not only in protein synthesis but also in protein degradation (caused by proteolytic venom components). The results are consistent with measured serum sIgG4 levels, which increased from 2.04 mgA/I at baseline to 7.25 mgA/I at 90 days. Moreover, the major proteomic changes were detected separately in the high responder group. This may suggest that changes in protein–peptide profiles reflect the actual response to VIT.

## 1. Introduction

Bees and wasps, part of the large *Hymenoptera* order, are abundant in every habitat where flowering plants occur. Therefore, stings caused by these insects are relatively common around the world [1]. Although most effects of a *Hymenoptera* sting observed in humans are mild to moderate, in sensitised individuals, the sting may result in serious allergic responses, including life-threatening anaphylaxis [2]. To protect against these dangerous reactions and to prevent morbidity and mortality from re-stings, venom immunotherapy (VIT) has been proposed. VIT, thus far the only disease-modifying treatment, is considered the most potent way to reduce the risk of anaphylaxis following bee and wasp stings [3,4].

Patients allergic to *Hymenoptera* venom with a history of anaphylactic reactions occurring after a sting constitute candidates for venom immunotherapy. Although the effectiveness of VIT is not questioned, the evidenced contraindications and risk of severe systemic side effects limit its administration [5]. Monitoring treatment progress may improve VIT safety. Monitoring is also a reasonable practice for predicting the success of VIT. According to the European Academy of Allergy and Clinical Immunology (EAACI), a gold standard for evaluating the success of the therapy is a sting challenge [6]. However, it is reliable only after reaching the maintenance dose [7] and cannot be used for checking the effects of the injected venom preparations in real time during treatment. Another important tool for the diagnosis of immediate hypersensitivity reactions is the basophil activation test (BAT). The BAT is a common method frequently used in diagnosing a venom response, and it is considered to be a standard approach in experimental studies [8,9]. Because of its high specificity and sensitivity, the BAT has also been successfully applied in VIT monitoring. According to the literature, a reduction in CD63-marked (activated) basophils has been observed during and after VIT [10,11,12]. Moreover, a study of the paediatric population revealed that the BAT allows for the identification of the culprit insect with higher specificity than the IgE reactivity test [11]. In addition, the high specificity of the BAT was demonstrated in *Hymenoptera*-venom-allergic patients with mastocytosis during VIT [13]. Hence, the BAT seems to be an optimal non-invasive tool for monitoring VIT. However, the EAACI does not recommend this method for VIT monitoring. Although basophil responses decrease markedly after VIT, which is likely associated with the induction of tolerance to *Hymenoptera* venom [14], according to the EAACI, performing the BAT does not allow one to estimate the individual risk for developing severe systemic reactions [6]. As noted in the recommendations, the BAT should be evaluated in order to be useful in the assessment of VIT’s clinical efficacy [6]. Thus, since the determination of venom-specific IgE and IgG levels, the allergen-blocking capacity or the BAT response is not recommended for VIT monitoring [6,15], implementing new therapy progress indicators poses an essential concern.

VIT’s immune mechanism is multifaceted. It is mainly related to the generation of allergen-specific regulatory T (Treg) and B (Breg) cell responses and, consequently, to the desensitisation of mast cells and basophils, a decrease in the activity of eosinophils and the regulation of IgE and IgG4 levels. These actions result in the suppression of cytokine and proliferative responses against venom allergens [16,17]. Although the cellular mechanism of VIT is quite well documented, little is known about the consequences of VIT for the proteome level. Therefore, the goal of this study was to investigate changes in protein–peptide patterns in *Hymenoptera*-venom-allergic patients undergoing VIT.

## 2. Materials and Methods

### 2.1. Characteristics of the Study Group

The study group consisted of 22 patients (aged 28 to 67 years) with a history of severe reaction to a *Hymenoptera* sting. According to the Mueller scale, grade III and grade IV severity was determined for 17 and 5 patients, respectively.

Sensitisation to the relevant *Hymenoptera* species was confirmed by measuring venom-specific IgE in serum (ImmunoCap ELISA; Phadia, Uppsala, Sweden) and via a positive skin prick test with venom allergens. The detailed data of sIgE and sIgG4 values are presented in Supplementary Materials in Spreadsheet S1 (sIgG4 and sIgE levels in the study group and the control group). It is worth noting that values obtained from ImmunoCap and other IgE tests (e.g., Immulite; Siemens Healthcare Diagnostics, Tarrytown, NY, USA) could not be substituted. However, a linear correlation has been observed between both assays [18]. Hence, the estimation of values from one assay for the other is possible, and this information is relevant for clinicians using assays other than ImmunoCap.

An allergy to wasp, bee and hornet venom was diagnosed for 16, 4 and 2 patients, respectively. The patients were qualified for the ultra-rush VIT protocol in accordance with the applicable standards—EAACI guidelines [19]. Following 1 day of the ultra-rush procedure, patients received maintenance doses of vaccines on day 11 and then, every 4 weeks.

The control group consisted of 7 patients (aged 29 to 61 years) allergic to *Hymenoptera* venoms but not undergoing immunotherapy. Of these, 5 patients did not consent to immunotherapy and 2 patients were not qualified for VIT due to low severity of sting-induced reactions (grade I according to Mueller).

Blood was taken 1 day before starting VIT, on the 1st day of the ultra-rush procedure (after the first doses of VIT) and then on the 11th, 90th and 180th day of immunotherapy. For patients not qualified for VIT (control group), blood was taken twice, 90 days apart.

The characteristics of the patients qualified for VIT (study group) and classified as the control group are presented in Table 1.

### 2.2. Measurement of sIgE and sIgG4 Serum Levels

sIgE and sIgG4 serum levels were measured using ImmunoCap (Phadia, Uppsala, Sweden) according to the manufacturer’s instructions. In brief, the tested antigens, covalently coupled to the solid phase, reacted with the specific IgE/IgG4 antibodies in the tested sample. Then, after washing, enzyme-labelled IgE/IgG4 antibodies were added to form a complex, which was subsequently incubated with a developing agent. After the reaction, the fluorescence of the eluate was measured, and then this was used to calculate the concentration of sIgE/IgG4. A calibration curve was used to evaluate the results. Specific IgE/IgG4 levels were estimated for wasp venom and honeybee venom.

### 2.3. Pre-Treatment of the Serum Samples

Purification, desalting and concentration of the proteomic fraction of the serum samples were performed using the ZipTip C18 (Millipore, Bedford, MA, USA) solid-phase extraction technique according to the manufacturer’s protocol. Acetonitrile (ACN) and a 0.1% solution of trifluoroacetic acid (TFA) in water were used for tips’ conditioning. Then, samples mixed with 0.1% TFA (1:5 ratio) were loaded onto the ZipTip pipette tips. Bound proteins and peptides, after washing in 0.1% TFA, were eluted with 4 µL of 50% acetonitrile (ACN) in 0.1% TFA.

### 2.4. MALDI-TOF MS Proteomic Profiling

Matrix-assisted laser desorption/ionisation–time-of-flight mass spectrometry (MALDI-TOF MS) protein–peptide profiling of ZipTip pre-treated serum samples was performed, as previously described [20]. In brief, 1 µL of each eluted fraction was mixed with 10 µL of 0.3 g/L α-cyano-4-hydroxycinnamic acid (HCCA) matrix solution in ethanol:acetone (2:1 ratio, *v*/*v*). These mixtures were spotted directly onto the AnchorChip Standard 800 µm (Bruker Daltonics, Bremen, Germany) target plate in triplicate. A MALDI-TOF/TOF UltrafleXtreme (Bruker Daltonics, Bremen, Germany) mass spectrometer was used for acquiring the spectra in MS-positive mode. The analysis range was set to mass-to-charge ratio (*m/z*) of 1000–10,000. This *m/z* range was considered optimal, as according to reports, on the one hand, an HCCA matrix background affects the lower range, and on the other, a decrease in the resolution in an *m/z* range of above 10,000 significantly impedes the detection of ions [21].

A mixture of Protein Calibration Standard I and Peptide Calibration Standard (Bruker Daltonics, Bremen, Germany) (5:1, *v*/*v*) was used for the external calibration. The average mass deviation from reference masses did not exceed 100 ppm. The MS parameters for the analysis were as follows: ion source 1, 25.09 kV; ion source 2, 23.80 kV; matrix suppression cut-off *m/z*, 700; pulsed ion extraction, 260 ns; and lens, 6.40 kV. From each spot, 2000 spectra (laser shots) were accumulated. FlexControl 3.4 (Bruker Daltonics, Bremen, Germany) software was applied for the acquisition and processing of MS spectra. Evaluation of intra-day and inter-day reproducibility and of the applied protocol was reported previously [22].

### 2.5. NanoLC-MALDI-TOF/TOF MS Identification of Discriminative Proteomic Features

Identification of the discriminative peaks between the studied groups was performed using a nano-liquid chromatography–matrix-assisted laser desorption/ionisation–time-of-flight/time-of-flight mass spectrometry (nanoLC-MALDI-TOF/TOF MS) system. Four microlitres of the eluent obtained via ZipTip sample depletion was subjected to nanoLC separation. HyStar 3.2 (Bruker Daltonics, Bremen, Germany) software was used for controlling the nanoLC set, which consisted of an EASY-nLC II (Bruker Daltonics, Bremen, Germany) nanoflow HPLC system and a Proteineer-fc II (Bruker Daltonics, Bremen, Germany) fraction collection device. The nanoLC system parts were an NS-MP-10 BioSphere C18 (NanoSeparations, Nieuwkoop, The Netherlands) trap column (20 mm × 100 µm I.D., particle size 5 µm, pore size 120 Å) and an Acclaim PepMap 100 (Thermo Scientific, Sunnyvale, CA, USA) column (150 mm × 75 µm I.D., particle size 3 µm, pore size 100 Å). The flow rate for the separation was 300 nL/min, and the linear gradient elution method was set to 2%–50% of ACN in 96 min (mobile phase A, 0.05% TFA in water; mobile phase B, 0.05% TFA in 90% ACN). Next, 22 min before gradient initiation, 80 µL of each of the 384 nanoLC fractions was mixed with 420 µL of the HCCA matrix solution (36 µL of HCCA saturated solution in 0.1% TFA and acetonitrile (90:10 ratio, *v*/*v*), 748 µL of acetonitrile and 0.1% TFA (95:5 ratio, *v*/*v*) mixture, 8 µL of 10% TFA and 8 µL of 100 mM ammonium phosphate) and automatically spotted onto the AnchorChip Standard 800 µm (Bruker Daltonics, Bremen, Germany) target plate. MS analysis was performed using an UltrafleXtreme (Bruker Daltonics, Bremen, Germany) tandem mass spectrometer working in positive MS/MS mode and a mass range of *m/z* of 700–3500. Per spot, 4000 spectra were accumulated. External calibration was performed using a Peptide Calibration Standard (Bruker Daltonics, Bremen, Germany) mixture with an average mass deviation of less than 1 ppm. The following settings were applied for MS and MS/MS modes: ion source 1, 7.50 kV; ion source 2, 6.75 kV; mass reflectron 1, 29.50 kV; mass reflectron 2, 14.00 kV; lens, 3.50 kV; lift 1, 19.00 kV; lift 2, 3.00 kV; and pulsed ion extraction time, 80 ns. WARP-LC (Bruker Daltonics, Bremen, Germany) software was used to establish a list of ions for identification following a screening of all the spots in reflector mode. For spectra acquisition, data processing and evaluation, FlexControl 3.4, FlexAnalysis 3.4 and BioTools 3.2 (Bruker Daltonics, Bremen, Germany) software were used. A SwissProt database and a Mascot 2.4.1 search engine with taxonomic restriction to Homo sapiens (humans) were applied for the identification of discriminative proteomic features. The protein search parameters were set to 1 missing cleavage, fragment ion mass tolerance *m/z* ± 0.7, precursor ion mass tolerance ±35 ppm, peptide charge +1, monoisotopic mass, carbamidomethyl (C) as fixed modification and oxidation (M), acetyl (N-term) and Glu→pyro-Glu (N-term E) as variable modifications.

### 2.6. Data Analysis

Processing of the acquired MS data was performed in an R software environment. MALDIquant and MALDIquantForeign packages were implemented. The Savitzky–Golay algorithm was applied for the smoothing of raw spectra. The statistics-sensitive non-linear iterative peak-clipping (SNIP) algorithm was used for baseline subtraction. Further processing of the spectra included calibration to the total ion current (TIC), estimation of the noise of mass spectrometry by median absolute deviation (MAD) function and spectra aligning to a signal-to-noise ratio of ≥2 (tolerance of 0.002). MetaboAnalyst 4.0 software was used for statistical analysis of the pre-processed MS patterns. Non-parametric statistical tests provided by MetaboAnalyst software were applied to compare studied groups. *p*-Values were calculated for the Wilcoxon rank-sum non-parametric test. False discovery rate (FDR) values were calculated based on the Benjamini–Hochberg procedure. Calculated *p*-values based on a *t*-test of <0.05 and false discovery rate (FDR) values of <0.05 were considered to be statistically significant. All the samples were analysed in random order and in triplicate. Hence, the spectra were grouped, and data obtained from corresponding repetitions were levelized and analysed as one biological replicate.

**Figure 1:**
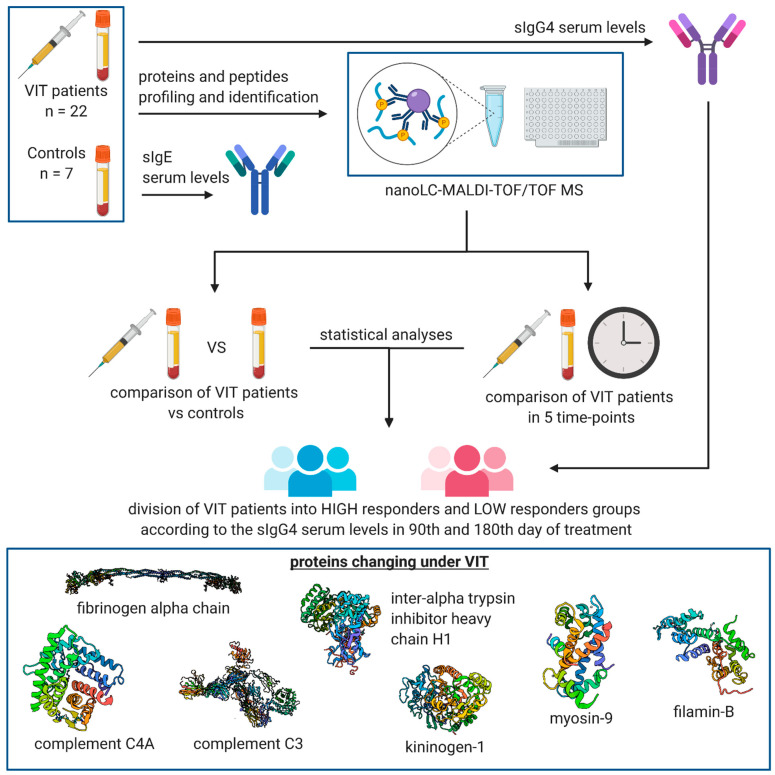
Workflow of the study (created with BioRender.com); VIT—venom immunotherapy; nanoLC-MALDI-TOF/TOF MS—nano-liquid chromatography–matrix-assisted laser desorption/ionisation–time-of-flight/time-of-flight mass spectrometry.

The schematic workflow of the study is presented in Figure 1.

## 3. Results

### 3.1. Serum sIgG4 Levels before and on the 90th and 180th Days of VIT

The mean value of serum sIgG4 concentrations increased from 2.04 mgA/I at baseline to 7.25 mgA/I at 90 days; then it slightly decreased to 6.85 mgA/I on the 180th day of treatment. These changes occurring during therapy were statistically significant: for the paired comparison analyses of one day before vs. 90th day of VIT and one day before vs. 180th day of VIT, the calculated *p*-values based on the *t*-test were both 4.2963 × 10^−5^. The false discovery rate (FDR) values were 0.00056732 and 0.0016755, respectively. Following VIT, significant variability in the individual IgG4 increase was observed among patients.

For the control allergic patients not treated with VIT, no differences between serum IgG4 levels were found at the two time-points.

### 3.2. MALDI-TOF MS Protein–Peptide Profiling and Identification of the Discriminatory Features

MALDI-TOF MS analyses results are presented in detail in Supplementary Materials in Document S1. The list of the relative intensities of all protein–peptide features detected in all samples is presented in Supplementary Materials in Spreadsheet S2. Briefly, the paired comparison analyses of serum protein–peptide patterns derived from patients undergoing VIT revealed changes in expressed proteins involving the fibrinogen alpha chain, complement C4-A, complement C3 and filamin-B. Compared to the controls, the fibrinogen alpha chain, complement C3, kininogen-1, myosin-9 and inter-alpha-trypsin inhibitor heavy chain H1 were altered.

**Figure 2:**
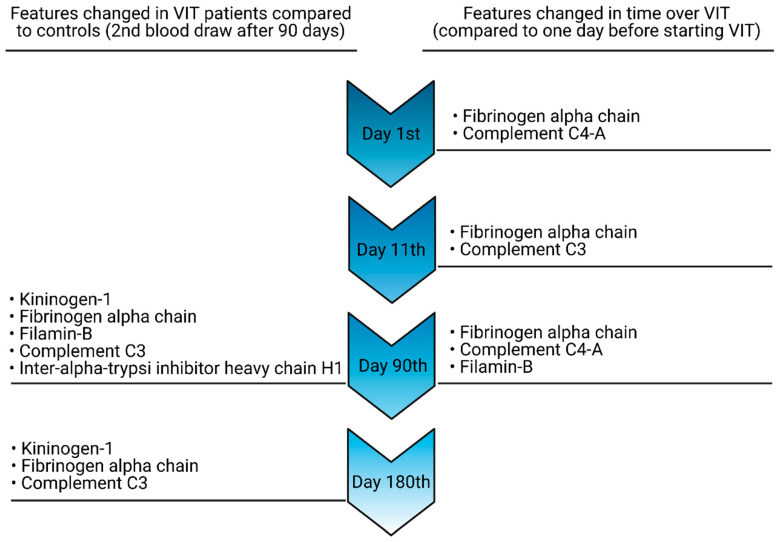
Proteomic features changing over VIT as compared to those at selected time-points with controls (left side) and patients one day before starting the treatment (right side).

Peaks changing over time, along with their identification and statistical significance, are summarised in Table 2 and Table 3 and also in Figure 2. Table 1 presents *m/z* features changed in MS spectra obtained from allergic patients undergoing VIT on the 1st, 11th and 90th days of treatment compared to samples collected from the same patients one day before starting VIT. Table 2 presents *m/z* features changing in MS spectra obtained from allergic patients undergoing VIT on the 90th and 180th days of VIT compared to samples collected from controls (second blood draw 90 days after the first one). Values of statistical parameters (*p*-value and FDR) calculated for each day are shown in columns. Statistically significant *p*-values and FDR values corresponding to proteins changed on a selected day are in bold. As statistically altered, we considered precursor ions for which both *p*-values and FDRs were <0.05. *p*-Values were calculated for the Wilcoxon rank-sum non-parametric test. The false discovery rate (FDR) is an additional parameter, which is suitable for multiple testing. It represents a percentage indicating the expected false positives among all features predicted to be significant. We decided to use the FDR parameter to enhance the statistical power of the test.

### 3.3. Comparison of Protein–Peptide Profiles Derived from Allergic Patients Undergoing VIT Classified as Low and High IgG4 Responder Groups

Referring to the observed individual variability in sIgG4 increase during VIT, patients were divided into two groups: low responders and high responders. The division was made separately for sIgG4 levels measured one day before, on the 90th and the 180th day of treatment. For a patient to qualify for the low responder group, two criteria had to be met simultaneously:

- The ratio of serum sIgG4 levels at two time-points < 15 (sIgG4 value of the 90th/180th day divided by the sIgG4 value of baseline)
- Differences between sIgG4 on the 90th/180th day and baseline < 6

After 90 days of VIT, 7 individuals were classified as low responders and 15 as high responders. After 180 days, 10 patients were classified as low responders and 12 as high responders.

The paired comparison analysis of protein–peptide patterns revealed that the majority of time-based changes come up in individuals classified as high responders. All discriminative peptides were identified as parts of the fibrinogen alpha chain, complement C3, kininogen-1, inter-alpha-trypsin inhibitor heavy chain H1, filamin-B, myosin-9 and complement C4-A. The data are shown in Supplementary Materials in Spreadsheet S3.

## 4. Discussion

The MS-based methodology and the paired statistical analysis applied in this study allowed for the identification of seven proteins significantly changed by the VIT process. For the first time, we identified the fibrinogen alpha chain, complement C3, complement C4A, inter-alpha trypsin inhibitor heavy chain H1, filamin-B, myosin-9 and kininogen-1 as important indicators of the progress of VIT. Peptides identified as parts of these above-mentioned proteins were detected in MS experiments with different intensities, depending on the day of treatment. The changes in MS spectra may suggest an altered expression of the identified proteins during VIT. Hence, since the depicted proteins are known to be involved in an immune inflammatory response, the results of our study mean that VIT induces such a response. The effects of VIT include activation of metabolic pathways, the complement cascade in particular. The complement system is part of the immune system, and it promotes inflammation. Thus, the activation of the complement pathway during VIT suggests triggering of the allergic inflammatory response, which is similar to the reaction to an insect sting. However, as venom doses administered during VIT are lower than during an insect sting, the allergic reaction is less outlined but leads to the development of adaptive immunity.

The differences occurring in the protein–peptide profiles were measurable from the very beginning of the therapy, and the number of *m/z* features increased in time up to the 90th day. With the first dose of the administered venom, the expression of three peptides changed. Two of them were identified as parts of fibrinogen alpha chain and one as fragment of complement C4A. However, *m/z* of 3315.61 remained unidentified. The inability to identify it may stem from the presence of neighbouring peaks or reduced peptide fragmentation. Moreover, the intact proteins and peptides detected in linear mode and statistically classified as discriminative between study groups must be subjected to identification undigested. It may impede the analysis as protein databases often include sequences obtained after enzymatic digestion, with arginine and lysine residues in the C-terminal and N-terminal regions. Therefore, identification of *m/z* of 3315.61 requires further investigation.

Comparisons at one day before starting VIT and on the 11th and 90th days both resulted in the identification of the fibrinogen alpha chain and complement C3. Additionally, in the latter comparison, filamin-B was identified. However, in total, three peptides were significantly altered on day 11, whereas on the 90th day, that number reached 14. The increased number of identified *m/z* features may be associated with the dose of administered venom. A higher venom dose may elicit a stronger immune response. However, once the maximum dose has been reached, patients receive only a maintenance dose every 4 weeks. After 180 days of VIT, immunity should already be acquired, and then the administration of a maintenance dose does not cause an allergic inflammatory response or causes a significantly milder course thereof (decreased reaction severity is also expected after an insect sting). This may explain the lack of visible changes in patients’ sera on the 180th day of VIT. Moreover, since MALDI is not a quantitative method, but a qualitative one, we focused not on studies of concentrations but on the dynamic changes caused by VIT occurring in the proteome. The results obtained in this study may suggest that during VIT, there may occur processes involved not only in protein synthesis but also in protein degradation, which is why we observe more fragments.

**Figure 3:**
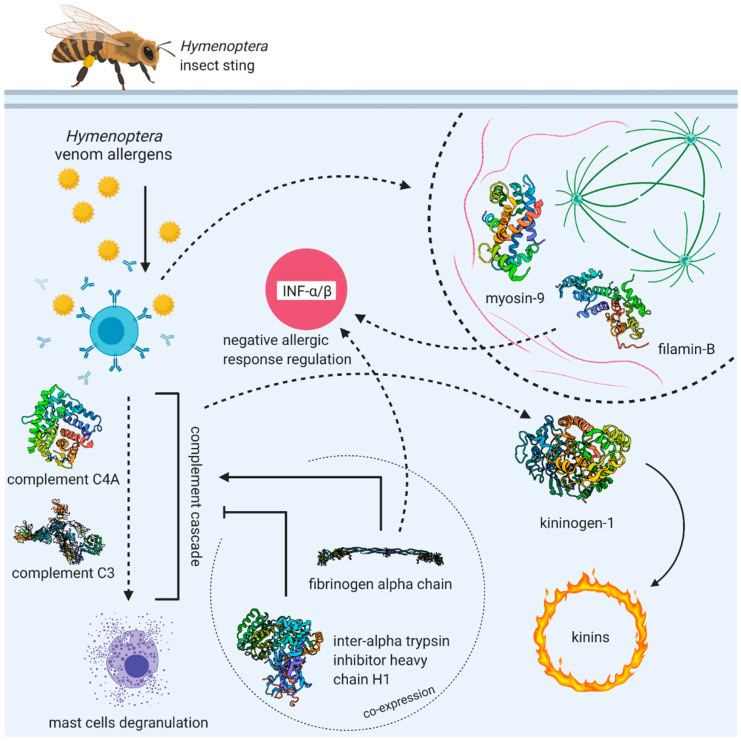
Interactions between identified proteins changing over VIT (created with BioRender.com); IFN-α/β—interferon-α/β.

A comparison of proteomic patterns in patients undergoing VIT and allergic individuals not qualified for the therapy resulted in the identification of kininogen-1, the fibrinogen alpha chain, myosin-9, complement C3 and inter-alpha-trypsin inhibitor heavy chain H1. These results confirm that VIT triggers biochemical processes, exemplified by measurable modifications in the expressed proteome. All of the proteins that this study has identified as being changed by the progress of VIT are associated with the inflammatory response (Figure 3). The changes are comparable to those occurring as a reaction to an insect sting [23].

Analyses were performed using a highly sensitive and specific MALDI-TOF mass spectrometry technique. To capture the dynamic proteome alterations occurring throughout desensitisation, samples were collected one day before starting the therapy and then, at four time-points: on the 1st, 11th, 90th and 180th days of treatment. However, we assumed that some differences might arise just from an allergic disorder and concomitant inflammation [23,24,25]. Hence, to corroborate our results, we analysed proteomic patterns obtained at two time-points (second blood collection 90 days after the first one) from individuals also sensitised to *Hymenoptera* venom but not qualified for VIT. Controls were not qualified for VIT because they did not agree to VIT or they had large local reactions (LLRs) after a sting and no systemic symptoms. The comparison of the MS data obtained from both studied groups enabled reliable verification of the variations at the proteomic level during VIT.

Fibrinogen is a soluble precursor of fibrin, known as a molecular indicator of an inflammatory response. It was found to activate macrophage chemokine secretion, cytokine secretion and leukocyte adhesion [26,27,28]. Hence, fibrinogen may be considered as a hallmark in a number of inflammatory diseases, including allergic asthma, allergic rhinitis and *Hymenoptera* venom allergy [23,29,30]. Participating in clot formation, fibrinogen limits the spread of inflammation and tempers its effects [31]. Moreover, its antioxidant activity eliminates the effects of oxidative stress. In that way, fibrinogen exhibits a protective role in an inflammatory response [32].

Importantly, fibrinogen is one of the main targets of insect venom proteases [33]. Proteases, including serine proteases, along with other enzymes, are an important fraction of *Hymenoptera* venom [34,35]. Bee venom serine protease (Bi-VSP), similarly to snake venom serine proteases, exhibits fibrino(geno)lytic activity. By activating prothrombin, proteases directly degrade fibrinogen [33,36]. Therefore, changes in fibrinogen levels found in this study may not only result from an inflammatory allergic reaction following exposure to venom but also be directly associated with the activity of venom compounds administered during VIT protocol.

**Figure 4:**
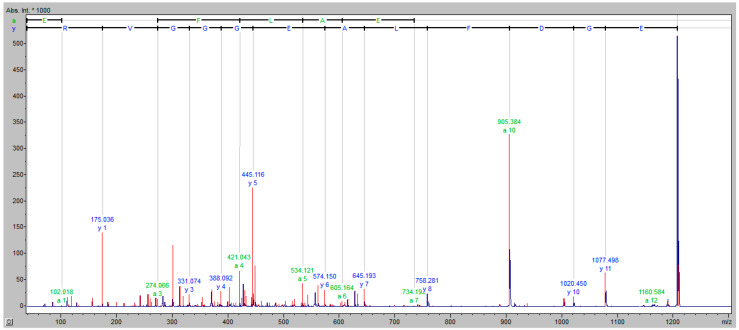
Fragmentation spectrum of peak *m/z* of 1207.25, identified as a fragment of the fibrinogen alpha chain; Y axis: absolute intensity x 1000; X axis: mass-to-charge ratio (*m/z*).

Figure 4 shows a representative fragmentation spectrum of peak *m/z* of 1207.25 identified as a fragment of the fibrinogen alpha chain, with a significant Mascot hit (80) based on the following peptide fragmentation sequence: G.EGDFLAEGGGVR.G.

Fibrinogen and other coagulation factors activate the complement cascade [37]. The complement system promotes the humoral immune response, resulting in the production of antibodies against foreign substances in order to eliminate them from the organism [38]. The importance of the complement system as a mediator of inflammation is related to the regulation of its various steps—complement components have the ability to stimulate cells involved in the immune-inflammatory response [39]. In this study, we detected complement C3 and complement C4-A as being a part of a complement pathway. It is interesting that the cleavage of complement components was demonstrated in Bothrops venom studies [40]. Therefore, probably similar mechanisms of activation of the complement system may occur after administration of different venoms from various organisms.

Overexpression of complement components may lead to an intensification of the processes within the complement pathway, causing an exacerbation of the allergic inflammatory reaction. The substantial protein instrumental in binding the complement components and, thereby, hindering their effects is inter-alpha-trypsin inhibitor heavy chain H1 (ITIH1). This acute-phase plasma glycoprotein is capable of attenuating either a classical or an alternative complement cascade [41,42]. In this study, we indicated the discriminatory potential of ITIH1 in comparison with individuals treated with VIT and allergic controls. This protein, alongside complement components and fibrinogen, was also found in our previous study as a prospective feature indicating an allergy to *Hymenoptera* venom [23]. Our findings are in agreement with the literature data that show that venom proteases active on fibrinogen from different species are involved in the envenomation mechanism [43,44,45]. It seems that venom immunotherapy also influences the fibrino(geno)lysis process. The fact that there is some overlap between the results of this study and the previous one (compared *Hymenoptera*-venom-allergic patients with healthy controls), and that some proteins are changed either by the allergy to *Hymenoptera* venom or due to VIT administration, indicates that the same mechanisms are involved in an immunological reaction to the venom. Controlled venom exposure triggers the development of an inflammatory response that is comparable to the reaction to a field insect sting. Obviously, the response following VIT is usually much less outlined. However, cases of severe anaphylaxis resulting from VIT have been reported [46]. 

Filamin-B, another protein identified in this study, is a large dimeric cytoplasmic actin-binding protein. It is involved in the organisation of the dynamic structure of the actin cytoskeleton, cell migration, extracellular signal transduction and RNA binding [47,48,49]. Moreover, it participates in the cellular response to type I interferon-α/β (IFN-α/β). This function of filamin-B is especially interesting in terms of VIT, as immunotherapy was found to impact interferon type I signalling [50]. IFNs are critical immunity factors involved in the negative regulation of an allergic response [51]. However, type I IFNs may promote the apoptosis of cells through the c-Jun N-terminal kinase (JNK) pathway as a response to cellular stress accompanying inflammation. Filamin-B accelerates this process by promoting JNK activation [52]. However, the interferon-stimulated gene 15 (ISG15) modification of filamin-B downregulates the JNK pathway, protecting neighbouring cells [53,54]. The alterations in filamin-B expression found in this study confirm the significance of type I IFN signalling (regulated by filamin-B) in the development of an allergic response. The measured changes in filamin-B concentration may relate directly to the IFN pathway. However, the elevation of filamin-B concentration may also accompany the accomplished apoptosis, when intracellular proteins are released from dead cells [55]. Anyway, both possible reasons are consistent, confirming the role of filamin-B and type I interferon in immunotherapy.

Myosin-9 is the next protein of cytoskeletal origin identified in this study. It is crucial for the cellular processes requiring actin cytoskeleton translocation and generation of the chemo-mechanical force [56]. The important functions of this protein are cell migration, cell adhesion and signal transduction [57,58]. However, the potential role of myosin-9 during VIT may be explained by the results of a recent study by Liu et al. [59]. According to that research, myosin-9 participates in the negative regulation of the innate immune response and suppression of the inflammation through the non-muscle myosin heavy chain-IIA–DNAX activating protein of 12 kDa–spleen tyrosine kinase (NMHC-IIA–DAP12-Syk) pathway. Moreover, it has been suggested that myosin-9 binds to a soluble form of CD163 and inhibits T lymphocyte proliferation and, consequently, suppresses the allergic inflammatory response [60,61].

This study also identified kininogen, a precursor of kinins, as another protein closely related to the inflammatory response. It is a multifunctional protein participating in various pathophysiological processes, including inflammation [62,63]. Kinins, including bradykinin, kallidin and T-kinin, and their active metabolites, act through the activation of bradykinin B1 and B2 receptors. They are mediators in the development of inflammation and pain; they also elevate vascular permeability and exhibit vasodilative activity [64,65]. The presence of kininogen fragments in analysed samples may indicate the kininogenase activity of *Hymenoptera* venoms, which is not surprising due to the presence of serine protease in bee, wasp and hornet venoms. Such an effect has been observed, e.g., in *Thalassophryne nattereri* fish venom [66] and *Bitis gabonica* rhinoceros [67] venom studies.

Venom immunotherapy is a kind of treatment strategy in which very small amounts of insect venom are administered subcutaneously to allergic patients. Non-purified, purified and the so-called depot (purified aluminium hydroxide adsorbed) preparations of venom are commercially available in Europe and the United States [68]. Regardless of the type and source, all preparations are based on natural venom extracts [69]. Hence, they may influence the human body in an analogous way to insect stings. According to the available literature, the development of an allergic reaction following a sting, in particular, causes the release of cellular inflammation components [70]. This fact has been proved in this study, as changes in inflammation mediator levels were found. Naturally, since the doses of the administered venom vary considerably compared to a real insect sting, the organism’s reaction is subtle and not all the proteomic components of a complete inflammation pathway were detected. However, the differences in protein–peptide profiles seem to get enhanced in time up to the 90th day of treatment. This may be associated with the incremental increase in the venom dose, as a higher dose of allergens stimulates a heavier allergic response.

Interestingly, differences have not been identified in the comparison of proteomic patterns derived from samples collected one day before and samples collected on the 180th day of VIT. This indicates the organism’s adaptation to venom allergens. A lack of measurable markers of an allergic response after venom administration may signal an increase in the organism’s toleration to allergens. Changes in the immunological system during immunotherapy are very dynamic. Proteomic profiles may also be different after subsequent months or years of VIT, and that requires further research.

Differences have not been identified either between protein–peptide profiles derived from allergic patients not qualified for VIT (control group) at two time-points or in the comparison of one day before versus the 1st day of therapy. However, some changes were measured on the 90th and 180th days compared to untreated allergic patients (see Table 2). These results confirm the specific proteomic alterations caused by desensitisation in time.

Although the effectiveness of VIT is relatively high, treatment failure is observed for a minor percentage of patients [71,72]. Since sIgG4 concentrations are reported to correlate with the progress and efficacy of desensitisation [73,74,75], to check whether serum protein–peptide patterns may predict the success of VIT, we correlated the proteomic data with the measured levels of sIgG4. Determination of specific IgG4 levels after 90 and 180 days of venom immunotherapy (VIT) made it possible to divide the patients into two groups: low responders and high responders. After 90 days of VIT, seven patients qualified for the low responder group. After 180 days of VIT, 10 patients qualified for the low responder group (7 persons were the same in both groups). Thus, seven patients had a consistent, persistent low increase in specific IgG4 after 180 days.

Possible causes of low response to VIT were analysed for these 7 patients. Two of them were desensitised with wasp venom, while it is known from clinical history that they had an anaphylactic shock after a hornet sting. Hypertension was a common trait of another three patients in the low responder group, while in the high responder group, there were no patients with cardiovascular disease. Patients with hypertension were treated with angiotensin-converting enzyme (ACE) inhibitors without B-blocker therapy. Therefore, it is possible that cardiovascular diseases and the drugs used for these diseases can affect not only the safety of the patients but also their immune response to the conducted VIT. However, we analysed a small group of patients. Therefore, the above statement should be confirmed in further studies and should not be overinterpreted at present.

Statistical analysis of proteomic data performed separately for low and high responder groups revealed changes occurring over time in the high responder groups (both divisions based on the sIgG4 levels on 90th and 180th days of VIT). Complete data are shown in Supplementary Materials, Spreadsheet S3. The number of discriminative peaks increased up to the 90th day. This correlation is consistent with the results obtained for the whole set of patients. In both divisions, the discriminative *m/z* precursor ions were identified as the fibrinogen alpha chain and complement C3. No changes were observed over time in the low responder group (sIgG4 on the 90th day). However, in the low responder group (according to sIgG4 levels on day 180), some differences occurred, but the number of changes was lower as compared to those in the high responders. Comparing high responders to controls, kininogen-1, the fibrinogen alpha chain, complement C3, complement C4A, inter-alpha-trypsin inhibitor heavy chain H1 and filamin-B have been identified.

The results of this study indicate the potential of proteomic analysis in the prediction of VIT efficacy. Importantly, such measurements may be performed right from the beginning of desensitisation and, thus, may enhance the safety of VIT. A lack of changes in the serum protein–peptide composition during treatment may flag a possible failure of treatment. Non-responding individuals may be referred for additional allergy tests. This may prevent serious allergic reactions in patients unaware of the lack of therapeutic effects of VIT.

## 5. Conclusions

Proteomic analysis may be the next step to personalised medicine, resulting in patients receiving therapy appropriate to their individual needs. In practice, we need diagnostic tools that can accurately indicate whether immunotherapy will end in success or failure. This will make it possible for some patients to avoid undergoing long-term and high-risk therapy and for us to focus on exploring new therapeutic solutions—for example, new VIT protocols. This topic certainly requires further research. Since MALDI-TOF MS is a qualitative method and the results of this study should be considered to be preliminary, complementary qualitative and transcriptomic analyses are planned to be performed in the next step.

## Figures and Tables

**Table 1:** Characteristics of the subjects.

Patient Number	Age (Years)	Insect That Causes an Allergic Reaction	Grade of the Allergic Reaction according to the Mueller Scale	Additional Diseases
**Study Group**				
UR-1	64	Wasp	IV	Hypertension
UR-2	58	Wasp	III	Allergic rhinitis, asthma
UR-3	38	Wasp	III	Asthma
UR-4	28	Wasp	III	Asthma
UR-5	31	Wasp	III	-
UR-7	64	Wasp	III	Asthma, hypertension
UR-8	54	Wasp	III	Allergic rhinitis, asthma
UR-9	67	Wasp	IV	Hypertension
UR-10	52	Wasp	III	Tachycardia
UR-11	57	Wasp	III	-
UR-19	48	Wasp	III	-
UR-28	51	Bee	III	-
UR-29	62	Hornet	IV	-
UR-31	50	Hornet	IV	Hypertension
UR-32	47	Bee	III	-
UR-33	42	Wasp	IV	-
UR-34	37	Wasp	III	Asthma
UR-35	45	Bee	III	-
UR-36	53	Wasp	III	-
UR-37	40	Bee	III	-
UR-38	46	Wasp	III	-
UR-40	40	Wasp	III	Allergic rhinitis, asthma
**Control Group**				
UR-13	29	Wasp	III	Allergic rhinitis
UR-14	30	Wasp	I	-
UR-20	48	Wasp	III	-
UR-21	61	Wasp	IV	Hypertension
UR-23	40	Wasp	III	-
UR-24	51	Wasp	III	Allergic rhinitis, hypertension, ischemic heart disease
UR-25	36	Wasp	I	Allergic rhinitis, hypothyroidism

**Table 2:** Peaks classified as discriminative between baseline (one day before starting treatment) and the 1st, 11th and 90th days of VIT.

Precursor Ion *m/z*	Protein Name	1st Day		11th Day		90th Day	
		*p*-Value	FDR	*p*-Value	FDR	*p*-Value	FDR
1617.57	Fibrinogen alpha chain	**0.00080919**	**0.029131**	>0.05	>0.05	**0.0010915**	**0.0056133**
1449.73	Complement C4-A	**0.0019183**	**0.03357**	>0.05	>0.05	>0.05	>0.05
2554.56	Fibrinogen alpha chain	**0.0027975**	**0.03357**	>0.05	>0.05	>0.05	>0.05
3315.61	x	**0.0042908**	**0.038618**	>0.05	>0.05	**0.018102**	**0.047737**
1077.93	Fibrinogen alpha chain	>0.05	>0.05	**0.0018253**	**0.044054**	**0.00084769**	**0.0050861**
1020.92	Fibrinogen alpha chain	>0.05	>0.05	**0.0031328**	**0.044054**	**0.00072854**	**0.0050861**
1519.87	Complement C3	0.030118	>0.05	**0.0036712**	**0.044054**	**0.00012064**	**0.0014477**
1466.66	Fibrinogen alpha chain	0.04616	>0.05	>0.05	>0.05	**1.1921 × 10^−5^**	**0.00028324**
1207.25	Fibrinogen alpha chain	>0.05	>0.05	>0.05	>0.05	**1.5736 × 10^−5^**	**0.00028324**
1351.37	Fibrinogen alpha chain	0.031164	>0.05	>0.05	>0.05	**0.0005094**	**0.0045846**
2093.20	Fibrinogen alpha chain	>0.05	>0.05	>0.05	>0.05	**0.0035243**	**0.015859**
4053.21	*m/z* > 3500	>0.05	>0.05	>0.05	>0.05	**0.0042908**	**0.017163**
1537.73	Fibrinogen alpha chain	0.011473	>0.05	>0.05	>0.05	**0.008316**	**0.029938**
1262.25	Fibrinogen alpha chain	0.018957	>0.05	>0.05	>0.05	**0.014095**	**0.042284**
2660.77	Fibrinogen alpha chain	>0.05	>0.05	>0.05	>0.05	**0.014095**	**0.042284**
1420.10	Filamin-B	>0.05	>0.05	>0.05	>0.05	**0.018564**	**0.047737**

FDR—false discovery rate; bold-statistically significant values.

**Table 3:** Peaks classified as discriminative between patients undergoing VIT (on the 90th and 180th day of treatment) and controls (second blood draw).

Precursor Ion *m/z*	Protein Name	90th Day		180th Day	
		*p*-Value	FDR	*p*-Value	FDR
1945.24	Kininogen-1	**0.000254**	**0.009156**	**0.000618**	**0.01857**
2093.20	Fibrinogen alpha chain	**0.000584**	**0.009573**	**0.001032**	**0.01857**
2660.77	Fibrinogen alpha chain	**0.000798**	**0.009573**	**0.008021**	**0.035102**
1546.56	Fibrinogen alpha chain	**0.001546**	**0.012693**	**0.00605**	**0.035102**
1331.52	Myosin-9	**0.001806**	**0.012693**	>0.05	>0.05
3315.61	x	**0.002116**	**0.012693**	**0.005189**	**0.035102**
4053.21	*m/z* > 3500	**0.004214**	**0.018932**	**0.008776**	**0.035102**
1519.87	Complement C3	**0.004545**	**0.018932**	**0.008021**	**0.035102**
1262.25	Fibrinogen alpha chain	**0.005259**	**0.018932**	**0.006973**	**0.035102**
1537.73	Fibrinogen alpha chain	**0.005259**	**0.018932**	**0.006973**	**0.035102**
1207.25	Fibrinogen alpha chain	**0.006998**	**0.022903**	**0.013758**	**0.045027**
1466.66	Fibrinogen alpha chain	**0.009238**	**0.027159**	**0.012058**	**0.04341**
1866.3	Complement C3	**0.009807**	**0.027159**	0.018957	>0.05
1221.15	Inter-alpha-trypsin inhibitor heavy chain H1	**0.016742**	**0.043051**	0.046026	>0.05
1020.92	Fibrinogen alpha chain	**0.019475**	**0.046741**	0.022328	>0.05
3240.91	Fibrinogen alpha chain	**0.022615**	**0.048532**	0.04588	>0.05
1617.57	Fibrinogen alpha chain	**0.022918**	**0.048532**	0.046123	>0.05

Bold-statistically significant values.

## Data Availability

The data presented in this study are available in Spreadsheet S1: sIgG4 and sIgE levels in study group and control group; Spreadsheet S2: List of relative intensities of proteomic features detected in all samples.

## Supplementary Materials

The following are available online at https://www.mdpi.com/2076-393X/9/3/249/s1: Spreadsheet S1: sIgG4 and sIgE levels in study group and control group; Spreadsheet S2: List of relative intensities of proteomic features detected in all samples; Spreadsheet S3: Low and high responders results; Document S1: Results.
