# Supplementary material for: Association between Venom Immunotherapy and Changes in Serum Protein—Peptide Patterns
Source: Vaccines (Basel). 2021 Mar 12;9(3):249. doi: 10.3390/vaccines9030249 (PMC8001044; doi:10.3390/vaccines9030249)
Supplement: Supplementary file 1 [file vaccines-09-00249-s001.zip › vaccines-1117847-revised sup/Supplementary Materials - Document S1 - Results.docx]

Supplementary Materials – Document S1: Results

## Comparison of protein-peptide profiles derived from allergic patients undergoing VIT one day before starting and on the 1^st^ day of treatment

Paired comparison analysis of MALDI-TOF MS profiles characteristic to patients one day before and on the 1^st^ day of VIT resulted in the selection of four peaks distinguishing between these groups (Table S1). The proposed nanoLC-MALDI-TOF/TOF MS strategy allowed for the identification of three of them. The identified proteins were fibrinogen alpha chain (FIBA_HUMAN) and complement C4-A (CO4A_HUMAN). However, the peak of m/z 3315.61 could not be identified, probably because of the presence of neighbouring peaks or due to poor fragmentation.

**Supplementary Table S1.** Characteristics of peptides discriminative between serum proteomic patterns derived from Hymenoptera venom allergic patients one day before starting VIT and on the 1st day of treatment

| Precursor  ion m/z | p-value | FDR | Peptide fragmentation  sequence | UniProtKB-ID | Protein name |
| --- | --- | --- | --- | --- | --- |
| 1617.57 | 0.00080919 | 0.029131 | T.ADSGEGDFLAEG GGVR.G | FIBA_HUMAN | Fibrinogen alpha chain |
| 1449.73 | 0.0019183 | 0.03357 | K.SHALQLNNRQIR.G | CO4A_HUMAN | Complement C4-A |
| 2554.56 | 0.0027975 | 0.03357 | K.SSSYSKQFTSSTSYN RGDSTFES.K | FIBA_HUMAN | Fibrinogen alpha chain |
| 3315.61 | 0.0042908 | 0.038618 | x | x | x |

## Comparison of protein-peptide profiles derived from allergic patients undergoing VIT one day before starting and on the 11th day of treatment

Peaks of m/z 1077.93 and 1020.92, distinguishing between proteomic patterns of allergic patients undergoing VIT one day before starting and on the 11th day of treatment were identified as fibrinogen alpha chain (FIBA_HUMAN). The analysis of a precursor ion m/z 1519.87 resulted in the identification of complement C3 (CO3_HUMAN). The data is shown in Table S2.

**Supplementary Table S2.** Characteristics of peptides discriminative between serum proteomic patterns derived from Hymenoptera venom allergic patients one day before starting VIT and on the 11th day of treatment

| **Precursor  ion m/z** | **p-value** | **FDR** | **Peptide fragmentation  sequence** | **UniProtKB-ID** | **Protein name** |
| --- | --- | --- | --- | --- | --- |
| 1077.93 | 0.0018253 | 0.044054 | E.GDFLAEGGGVR.G | FIBA_HUMAN | Fibrinogen alpha chain |
| 1020.92 | 0.0031328 | 0.044054 | G.DFLAEGGGVR.G | FIBA_HUMAN | Fibrinogen alpha chain |
| 1519.87 | 0.0036712 | 0.044054 | G.SPMYSIITPNILR.L  (+ Oxidation) | CO3_HUMAN | Complement C3 |

## Comparison of protein-peptide profiles derived from allergic patients undergoing VIT one day before starting and on the 90th day of treatment

Fourteen peaks have been classified as discriminative between serum proteomic profiles characteristic to *Hymenoptera* venom allergic patients one day before starting VIT and on the 90th day of treatment (Table S3). Ten of them have been identified as fibrinogen alpha chain (FIBA_HUMAN), one as complement C3 (CO3_HUMAN), and one as filamin-B (FLNB_HUMAN). However, two peaks of m/z; 4053.21; and 3315.61 could not be identified and require further investigation.

**Supplementary Table S3.** Characteristics of peptides discriminative between serum proteomic patterns derived from Hymenoptera venom allergic patients one day before starting VIT and on the 90th day of treatment

| **Precursor  ion m/z** | **p-value** | **FDR** | **Peptide fragmentation  sequence** | **UniProtKB-ID** | **Protein name** |
| --- | --- | --- | --- | --- | --- |
| 1466.66 | 1.1921 x10-5 | 0.00028324 | A.DSGEGDFLAEG GGVR.G | FIBA_HUMAN | Fibrinogen alpha chain |
| 1207.25 | 1.5736 x10-5 | 0.00028324 | G.EGDFLAEGGGVR.G | FIBA_HUMAN | Fibrinogen alpha chain |
| 1519.87 | 0.00012064 | 0.0014477 | G.SPMYSIITPNILR.L  (+ Oxidation) | CO3_HUMAN | Complement C3 |
| 1351.37 | 0.0005094 | 0.0045846 | D.SGEGDFLAEG GGVR.G | FIBA_HUMAN | Fibrinogen alpha chain |
| 1020.92 | 0.00072854 | 0.0050861 | G.DFLAEGGGVR.G | FIBA_HUMAN | Fibrinogen alpha chain |
| 1077.93 | 0.00084769 | 0.0050861 | E.GDFLAEGGGVR.G | FIBA_HUMAN | Fibrinogen alpha chain |
| 1617.57 | 0.0010915 | 0.0056133 | T.ADSGEGDFLAEG GGVR.G | FIBA_HUMAN | Fibrinogen alpha chain |
| 2093.20 | 0.0035243 | 0.015859 | A.DEAGSEADHEG THSTKRGHA.K | FIBA_HUMAN | Fibrinogen alpha chain |
| 4053.21 | 0.0042908 | 0.017163 | m/z > 3500 | m/z > 3500 | m/z > 3500 |
| 1537.73 | 0.008316 | 0.029938 | T.ADSGEGDFLAEG GGVR.G | FIBA_HUMAN | Fibrinogen alpha chain |
| 1262.25 | 0.014095 | 0.042284 | S.GEGDFLAEG GGVR.G | FIBA_HUMAN | Fibrinogen alpha chain |
| 2660.77 | 0.014095 | 0.042284 | A.DEAGSEADHEG THSTKRGHAK SRPV.R | FIBA_HUMAN | Fibrinogen alpha chain |
| 3315.61 | 0.018102 | 0.047737 | x | x | x |
| 1420.10 | 0.018564 | 0.047737 | K.VFGPGIEGKDVFR.E | FLNB_HUMAN | Filamin-B |

## Comparison of protein-peptide profiles derived from allergic patients undergoing VIT one day before starting and on the 180th day of treatment

Serum proteomic patterns derived from allergic patients classified to VIT one day before starting the treatment and on the 180th day did not significantly change in time.

## Comparison of protein-peptide profiles derived from allergic patients not qualified to VIT (control group) in two time-points

Paired comparison analysis of serum protein-peptide patterns obtained twice from Hymenoptera venom allergic patients not qualified to VIT (second serum collection 90 days after the first one) revealed no statistically significant differences.

## Comparison of protein-peptide profiles derived from Hymenoptera venom allergic patients one day before starting VIT (test group) and those of patients not qualified to VIT (control group – first serum collection)

Non-paired comparison analysis showed no differences between proteomic features obtained from allergic patients qualified to VIT one day before starting the treatment and allergic controls (first serum collection).

## Comparison of protein-peptide profiles derived from allergic patients on the 90th day of VIT (test group) and those of patients not qualified to VIT (control group – second serum collection 90 days after the first one)

The non-paired comparison analysis revealed changes occurring in protein-peptide profiles characteristic to patients undergoing VIT (on the 90th day of treatment) compared to *Hymenoptera* venom allergic controls (second serum collection 90 days after the first one). In total, seventeen precursor ions have been classified as discriminative between these groups (Table S4). Ten peaks have been identified as parts of the fibrinogen alpha chain (FIBA_HUMAN). Moreover, two peaks have been identified as complement C3 (CO3_HUMAN), one as kininogen-1 (KNG1_HUMAN), one as myosin-9, and one as inter-alpha-trypsin inhibitor heavy chain H1. Proteomic features of m/z 3315.61; and 4053.21 could not be detected and require further investigation.

**Supplementary Table S4.** Characteristics of peptides discriminative between serum proteomic patterns derived from Hymenoptera venom allergic patients qualified to VIT on the 90th day of treatment and from patients not qualified to treatment (second serum collection 90 days after the first one)

| **Precursor ion m/z** | **p-value** | **FDR** | **Peptide fragmentation sequence** | **UniProtKB-ID** | **Protein name** |
| --- | --- | --- | --- | --- | --- |
| 1945.24 | 0.00025434 | 0.0091561 | H.NLGHGHKHERDQG HGHQ.R | KNG1_HUMAN | Kininogen-1 |
| 2093.20 | 0.0005843 | 0.009573 | A.DEAGSEADHEGTH STKRGHA.K | FIBA_HUMAN | Fibrinogen alpha chain |
| 2660.77 | 0.00079775 | 0.009573 | A.DEAGSEADHEGTH STKRGHAKSRPV.R | FIBA_HUMAN | Fibrinogen alpha chain |
| 1546.56 | 0.0015457 | 0.012693 | A.DSGEGDFLAEGGG VR.G (+ Phospho (ST)) | FIBA_HUMAN | Fibrinogen alpha chain |
| 1331.52 | 0.0018058 | 0.012693 | R.QLEEAEEEAQR.A | MYH9_HUMAN | Myosin-9 |
| 3315.61 | 0.0021155 | 0.012693 | x | x | x |
| 4053.21 | 0.0042143 | 0.018932 | m/z > 3500 | m/z > 3500 | m/z > 3500 |
| 1519.87 | 0.0045449 | 0.018932 | G.SPMYSIITPNILR.L  (+ Oxidation) | CO3_HUMAN | Complement C3 |
| 1262.25 | 0.0052588 | 0.018932 | S.GEGDFLAEGGGVR.G | FIBA_HUMAN | Fibrinogen alpha chain |
| 1537.73 | 0.0052588 | 0.018932 | T.ADSGEGDFLAEG GGVR.G | FIBA_HUMAN | Fibrinogen alpha chain |
| 1207.25 | 0.0069982 | 0.022903 | G.EGDFLAEGGGVR.G | FIBA_HUMAN | Fibrinogen alpha chain |
| 1466.66 | 0.0092379 | 0.027159 | A.DSGEGDFLAEG GGVR.G | FIBA_HUMAN | Fibrinogen alpha chain |
| 1866.30 | 0.0098074 | 0.027159 | R.SSKITHRIHWES ASLL.R | CO3_HUMAN | Complement C3 |
| 1221.15 | 0.016742 | 0.043051 | R.GHMLENHVER.L | ITIH1_HUMAN | Inter-alpha-trypsin inhibitor heavy chain H1 |
| 1020.92 | 0.019475 | 0.046741 | G.DFLAEGGGVR.G | FIBA_HUMAN | Fibrinogen alpha chain |
| 3240.91 | 0.022615 | 0.048532 | K.SYKMADEAGSEADHE GTHSTKRG HAKSRPV.R | FIBA_HUMAN | Fibrinogen alpha chain |
| 1617.57 | 0.022918 | 0.048532 | T.ADSGEGDFLAEG GGVR.G | FIBA_HUMAN | Fibrinogen alpha chain |

## Comparison of protein-peptide profiles derived from allergic patients on the 180th day of VIT (test group) and those of patients not qualified to VIT (control group – second serum collection 90 days after the first one)

The comparison of protein-peptide profiles obtained from allergic patients qualified to VIT on the 180th day of treatment and allergic controls (second serum collection 90 days after the first one) allowed for the depiction of eleven peaks distinguishing between these groups (Table S5). The analysis of seven peaks resulted in the identification of the fibrinogen alpha chain (FIBA_HUMAN). Additionally, kininogen-1 (KNG1_HUMAN), and complement C3 (CO3_HUMAN) were identified. However, the identification of signals m/z 3315.61 and 2660.77 requires further analysis.

**Supplementary Table S5.** Characteristics of peptides discriminative between serum proteomic patterns derived from Hymenoptera venom allergic patients qualified to VIT on the 180th day of treatment and from patients not qualified to treatment (second serum collection 90 days after the first one)

| **Precursor ion m/z** | **p-value** | **FDR** | **Peptide fragmentation sequence** | **UniProtKB-ID** | **Protein name** |
| --- | --- | --- | --- | --- | --- |
| 1945.24 | 0.00061842 | 0.01857 | H.NLGHGHKHERDQ GHGHQ.R | KNG1_HUMAN | Kininogen-1 |
| 2093.20 | 0.0010317 | 0.01857 | A.DEAGSEADHEGTHST KRGHA.K | FIBA_HUMAN | Fibrinogen alpha chain |
| 3315.61 | 0.0051885 | 0.035102 | x | x | x |
| 1546.56 | 0.0060501 | 0.035102 | A.DSGEGDFLAEGGGVR.G  (+ Phospho (ST)) | FIBA_HUMAN | Fibrinogen alpha chain |
| 1262.25 | 0.006973 | 0.035102 | S.GEGDFLAEGGGVR.G | FIBA_HUMAN | Fibrinogen alpha chain |
| 1537.73 | 0.006973 | 0.035102 | T.ADSGEGDFLAEGGGVR.G | FIBA_HUMAN | Fibrinogen alpha chain |
| 1519.87 | 0.0080205 | 0.035102 | G.SPMYSIITPNILR.L  (+ Oxidation) | CO3_HUMAN | Complement C3 |
| 2660.77 | 0.0080205 | 0.035102 | A.DEAGSEADHEGTHST KRGHAKSRPV.R | FIBA_HUMAN | Fibrinogen alpha chain |
| 4053.21 | 0.0087755 | 0.035102 | m/z > 3500 | m/z > 3500 | m/z > 3500 |
| 1466.66 | 0.012058 | 0.04341 | A.DSGEGDFLAEGGGVR.G | FIBA_HUMAN | Fibrinogen alpha chain |
| 1207.25 | 0.013758 | 0.045027 | G.EGDFLAEGGGVR.G | FIBA_HUMAN | Fibrinogen alpha chain |
